# Metabolic changes in mice cardiac tissue after low-dose irradiation revealed by ^1^H NMR spectroscopy

**DOI:** 10.1093/jrr/rrz079

**Published:** 2019-12-16

**Authors:** Michalina Gramatyka, ᴌukasz Boguszewicz, Mateusz Ciszek, Dorota Gabryś, Roland Kulik, Maria Sokół

**Affiliations:** Maria Sklodowska-Curie Institute - Oncology Center, Gliwice Branch, Wybrzeże Armii Krajowej 15, 44-101 Gliwice, Poland

**Keywords:** ionizing radiation, cardiotoxicity, NMR spectroscopy, metabolome

## Abstract

Ionizing radiation may cause cardiotoxicity not only at high, but even at low (considered as harmless) doses, yet the molecular mechanisms of the heart’s response to low doses are not clear. In this work, we used high-resolution nuclear magnetic resonance (NMR) spectroscopy to detect the early and late effects of radiation on the metabolism of murine hearts. The hearts of C57Bl/6NCrl female mice were irradiated *in vivo* with single 0.2 Gy or 2 Gy doses using 6 MV photons, then tissues were collected 48 h and 20 weeks after exposure. The most distinct changes in the profile of polar metabolites were detected 48 h after irradiation with 2 Gy, and included increased levels of pantothenate and glutamate as well as decreased levels of alanine, malonate, acetylcarnitine, glycine and adenosine. Significant effects of the 2 Gy dose were also observed 20 weeks after irradiation and included decreased levels of glutamine and acetylcarnitine when compared with age-matched controls. Moreover, several differences were observed between hearts irradiated with 2 Gy and analyzed either 48 h or 20 weeks after the exposure, which included changes in levels of acetylcarnitine, alanine, glycine, glutamate, glutamine, formate, myo-inositol and trimethylamine. No statistically significant effects induced by the 0.2 Gy dose were observed 20 weeks after irradiation. In general, radiation-affected compounds were associated with energy metabolism, fatty acid beta-oxidation, oxidative stress and damage to cell structures. At the same time, radiation-related effects were not detected at the level of tissue histology, which indicated a higher sensitivity of metabolomics-based tests for cardiac tissue response to radiation.

## INTRODUCTION

Ionizing radiation can damage all types of cells, but the radiosensitivity of actively dividing cells is much higher than that of cells that are neither meiotically nor mitotically active. The mammalian heart has long been considered a post-mitotic organ implying that the total number of cardiomyocytes is set at birth. However, studies have shown that measurable myocyte turnover occurs in adult mammalian and human hearts, and mitogenic cardiomyocytes, with sensitivity characteristic for dividing cells, can appear in a heart after myocardial infarction [1].

It is known that a single exposure to a high dose of radiation (>15 Gy) delivered to the heart exerts an adverse long-term effect on cardiovascular functions. It results in morphological degeneration, mechanical dysfunction, damage to the endothelium and increased mortality. Such radiation-induced cardiac injuries are suggested to be mediated by a micro-vascular injury caused by inflammation and oxidative stress [2]. On the other hand, currently little is known about the heart’s response to low doses of ionizing radiation. In particular, the long-term consequences of such exposure are not well understood. A number of studies have suggested that the biological and molecular response to very low doses and dose rates of ionizing radiation may differ from that of high doses [3]. For instance, in endothelial cells low doses of X-rays (0.05 Gy) were found to induce apoptosis and a higher number of DNA double-strand breaks than higher doses (0.5 Gy) [4]. However, very low doses (0.025–0.05 Gy), given at a low dose rate, were shown to be protective [5].

Ionizing radiation, used in cancer therapy and other medical procedures, can increase the risk of cardiovascular diseases. In some cases, the adverse effect of radiation on the cardiovascular system can be observed a few hours after the irradiation, but the development of clinical symptoms of cardiotoxicity usually takes up to 30–40 years [6–8]. Depending on the parameters measured, various studies indicate that the risk of such late cardiovascular symptoms may affect up to 60% of patients whose hearts were exposed to radiation during radiotherapy [9]. Also, increased efficacy of cancer treatment results in the increased survival of cancer patients, which in turn leads to an increase in the frequency of detection of late side-effects of radiotherapy. Although some researchers suggest that low doses delivered to the heart are harmless and unlikely to increase cardiac mortality [9], a growing number of reports show a relationship between low radiation doses and the frequency of heart diseases. These studies indicate that clinically low doses (accumulated dose <4 Gy) may also increase the risk of a cardiac event (usually ischemic heart disease), even though the time between exposure and the occurrence of clinical symptoms can be very long [6,8,10]. Radiation-induced effects on the heart are distinctly non-linear with doses [11,12], thus extrapolations from high- to low-dose radiation effects do not seem to be fully applicable. As an increased risk cannot be evidenced by epidemiology alone at low doses, further *in vitro* and *in vivo* mechanistic studies focused on the elucidation of molecular pathways are needed.

High-resolution nuclear magnetic resonance (NMR) spectroscopy is a powerful and well-established technique for the detection of metabolites in complex biological samples e.g. serum, urine, tissues or tissue extracts. NMR was successfully used to study radiation-induced alterations in humans, including in brain [13], serum [14] and feces [15], as well as in animals, including in serum and urine [16,17], bone [18] and spleen [19]. However, literature regarding metabolic changes specifically in the heart is sparse [20].

We have previously shown that exposure of cardiomyocytes *in vitro* to a relatively low dose of radiation (2 Gy) that does not affect cell viability, markedly affects their metabolic profiles. High-resolution magic angle spinning NMR analysis performed in living cells 48 h after irradiation revealed changes in levels of metabolites associated mainly with oxidative stress (glutamate, GSH (glutathione reduced), taurine), perturbations in energy metabolism (valine, isoleucine, GSH, glycine, threonine, taurine), membrane metabolism (phospholipids, cholines, glycine, taurine) and amino acid metabolism [21].

In the present work, we aimed to investigate metabolic changes induced by radiation *in vivo*. We employed proton NMR (^1^H NMR) to analyze the early and late effects of radiation on metabolic profiles in extracts of heart tissue from mice irradiated with 0.2 and 2 Gy doses.

## MATERIALS AND METHODS

### Animals

In the experiment we used C57Bl/6NCrl female mice, housed in our animal facility in pathogen-free conditions (Specific Pathogen Free (SPF) standards). The study protocol was approved by the Committee on the Ethics of Animal Experiments of the Local Ethics Commission (Medical University of Silesia, Katowice, Poland).

The animals were divided into four groups (five animals in each group): (i) control, not irradiated mice; (ii) mice irradiated with a single 2 Gy dose 48 h before sample collection; (iii) mice irradiated with a single 2 Gy dose 20 weeks before sample collection; (iv) mice irradiated with a single 0.2 Gy dose 20 weeks before sample collection. All animals were sacrificed at the age of 28 weeks to avoid age-related differences between groups. Consequently, animals sacrificed 20 weeks after the exposure were irradiated at the age of 8 weeks, whereas animals sacrificed 48 h after the exposure were irradiated at the age of 28 weeks.

Before irradiation mice were anesthetized with Avertin (0,5 mg/kg). Irradiation was performed in our center using a Varian CLINAC 2300 linear accelerator, with 6 MV photons, the dose rate was set to 300 MU/min, as previously reported [22]. Before conducting experiments, the representative mice were Computed Tomography (CT) scanned and their hearts were visualized and contoured using the Eclipse system. Treatment plans were performed to calculate the exact number of MU needed to deliver the required dose to the heart when 50% of the dose was given from the 0 degree field and 25% from the side fields. The constant source to surface distance (SSD) was set to 100 cm and information obtained from treatment plans allowed the creation of the optimal dimension of three treatment fields of 1.5 x 1.5 cm. The exact position of the heart was assessed by radiography on the Acuity simulator shortly before irradiation.

Hearts were removed immediately after the animals were sacrificed and dissected in halves (vertically from the atrium to the apex of the heart) and were either snap-frozen in liquid nitrogen and stored at −80 °C until further analysis on NMR (five heart samples for each group) or fixed in formalin and embedded in paraffin for histology analysis of tissue sections (three heart samples for each group).

### Histological analyses

To assess changes in collagen content the Masson trichrome stain was performed according to the manufacturer’s instructions (ScyTek Laboratories, USA). In brief, tissue sections were deparaffinized, hydrated and then stained with Bouin’s fluid, Weigert’s iron hematoxylin, Biebrich Scarlet/acid fuchsin solution, phosphomolybdic/phosphotungstic acid solution, Aniline Blue solution and acetic acid solution, dehydrated and mounted in Dibutylphthalate Polystyrene Xylene (DPX) mountant. Slides were analyzed under a light microscope for changes in collagen content seen as changes in the intensity of blue staining.

To analyze changes in the number of apoptotic cells the Terminal deoxynucleotidyl transferase dUTP Nick End Labeling (TUNEL) assay was performed according to the manufacturer’s instructions (In Situ Cell Death Detection Kit, TMR red, Roche, Germany). In brief, tissue sections were deparaffinized and rehydrated. Afterward, we performed an antigen retrieval by boiling in 0.1 M citrate buffer, pH 6.0. The slides were labeled with TUNEL reaction mixture, rinsed and mounted in medium with 4’,6-diamidino-2-phenylindole (DAPI). Slides were analyzed under a fluorescence microscope, using blue fluorescence to visualize cell nuclei and red fluorescence to visualize apoptotic cells. TUNEL-positive nuclei were counted in whole sections.

### NMR sample preparation

Polar metabolites were extracted from the hearts for the metabolic studies in accordance with Beckonert’s procedure [23]. In brief, an ice-cold solution of methanol and water (in proportion 8:2.5) was added to the tubes with frozen heart samples. The tissue was homogenized on ice using a Hielscher UP50H ultrasonic processor using 30 s pulses over a period of 5 min. The samples were incubated on ice for 15 min and then centrifuged at 1000 × g for 15 min at 4°C. The supernatant was transferred to a new tube and the solvent was evaporated from the samples using a speed vacuum concentrator. The material was re-suspended in 580 μL of phosphate buffer, pH 7.4 (in D_2_O containing Trimethylsilylpropanoic acid (TSP) and NaN_3_). The samples were vortexed and centrifuged at 12.000 × g for 5 min and for each sample 550 μL of the supernatant was transferred into the NMR tube (Wilmad Labglass, USA) and kept at 4°C until the NMR analysis.

### NMR measurement protocols

^1^H NMR spectra were acquired on a Bruker 400.13 MHz Avance III spectrometer (Bruker Biospin, Rheinstetten, Germany) equipped with a 5 mm PABBI probe, located at the Department of Medical Physics, MSC Institute—Oncology Center, Gliwice Branch. Quality control tests were performed at the beginning of each measurement day. NMR probe tuning and matching, shimming, determination of the transmitter offset value for water pulse presaturation and 90^°^ pulse adjustments were always made for each sample. Receiver gain was set to 90.5 and temperature to 300 K for all experiments. Two types of ^1^H NMR spectra were acquired for each sample: NOESY (nuclear Overhauser effect spectroscopy) and JRES (J-resolved).

- NOESY spectra were used to obtain an overview of all types of molecules. The sequence parameters were as follows: pulseprogram noesygppr1d, time domain 64 k, number of scans 256, relaxation delay 4 s, mixing time 10 ms, spectral width 30 ppm, acquisition time 2.73 s, total scan time 29 min 20 s.
- 2D JRES spectra were used to visualize scalar couplings and improve metabolite identification, whereas 1D projections of JRES spectra were used in data analysis. The sequence parameters were as follows: pulseprogram jresgpprqf, time domain 8 k, number of scans per increment 16, number of dummy scans 16, number of increments 40, spectral widths 16 ppm (F2) and 78 Hz (F1), acquisition time 0.62 s, relaxation delay 2 s, total scan time 31 min 39 s.

The spectra were post-processed with line a broadening of 0.3 Hz and automatically phase-corrected (in Topspin software from Bruker Biospin), referenced to the methyl doublet of alanine at 1.48 ppm and bucketed over the region of 10.0–0.5 ppm with the bucket width set to 0.002 ppm using AMIX software (Bruker Biospin). The spectral regions of water (5.15–4.38 ppm, d = 0.77 ppm) and methanol (3.39–3.34 ppm, d = 0.05 ppm) were removed from the analysis in order to prevent variation in each sample. The spectra were normalized to the total area of the metabolites’ spectral integrals to compensate for possible differences in sample concentration.

### Metabolite identification and quantification

The metabolites were identified by comparing their chemical shifts with those for the standard compounds from Chenomx NMR Suite Professional database (Chenomx Inc., Edmonton, Canada). In case of assignment ambiguity spectral identification was supported with multiplicity and scalar couplings information from the 2D J-resolved spectra as well as the Human Metabolome Database and available literature.

1D projections of the JRES spectra were used for quantification of the metabolite signals. The peaks in the 1D spectra were integrated by area in AMIX software (Bruker, Biospin). The peak integrals were measured in the individually (i.e. for each metabolite separately) adjusted spectral regions.

### Statistical analyses

Data analysis was carried out using Statistica software (Statsoft v.12). The Mann-Whitney U test and Kruskal-Wallis ANOVA test were used for assessment of the statistical significance of differences in metabolite levels (*P*-values threshold < 0.05). Furthermore, a fold-change (FC) with standard error (SE) approach was used for additional visualization of the metabolic differences between the studied groups. The significance threshold according to FC was set as FC + SE < 1 (for FC < 1) or FC − SE > 1 (for FC > 1). Principal component analysis (PCA) was conducted in SIMCA software (Umetrics, v. 14).

## RESULTS

In this work, early and late effects of ionizing radiation on the metabolism of heart tissue were addressed using ^1^H NMR. Murine hearts irradiated with a single 0.2 Gy or 2 Gy dose were analyzed 20 weeks after exposure (to both doses) or 48 h after exposure (to the higher dose). Simultaneously, possible radiation-induced effects in tissue morphology (the content of collagen fibers and the number of apoptotic cells) were analyzed to provide context for a molecular picture.

**Fig. 1.**
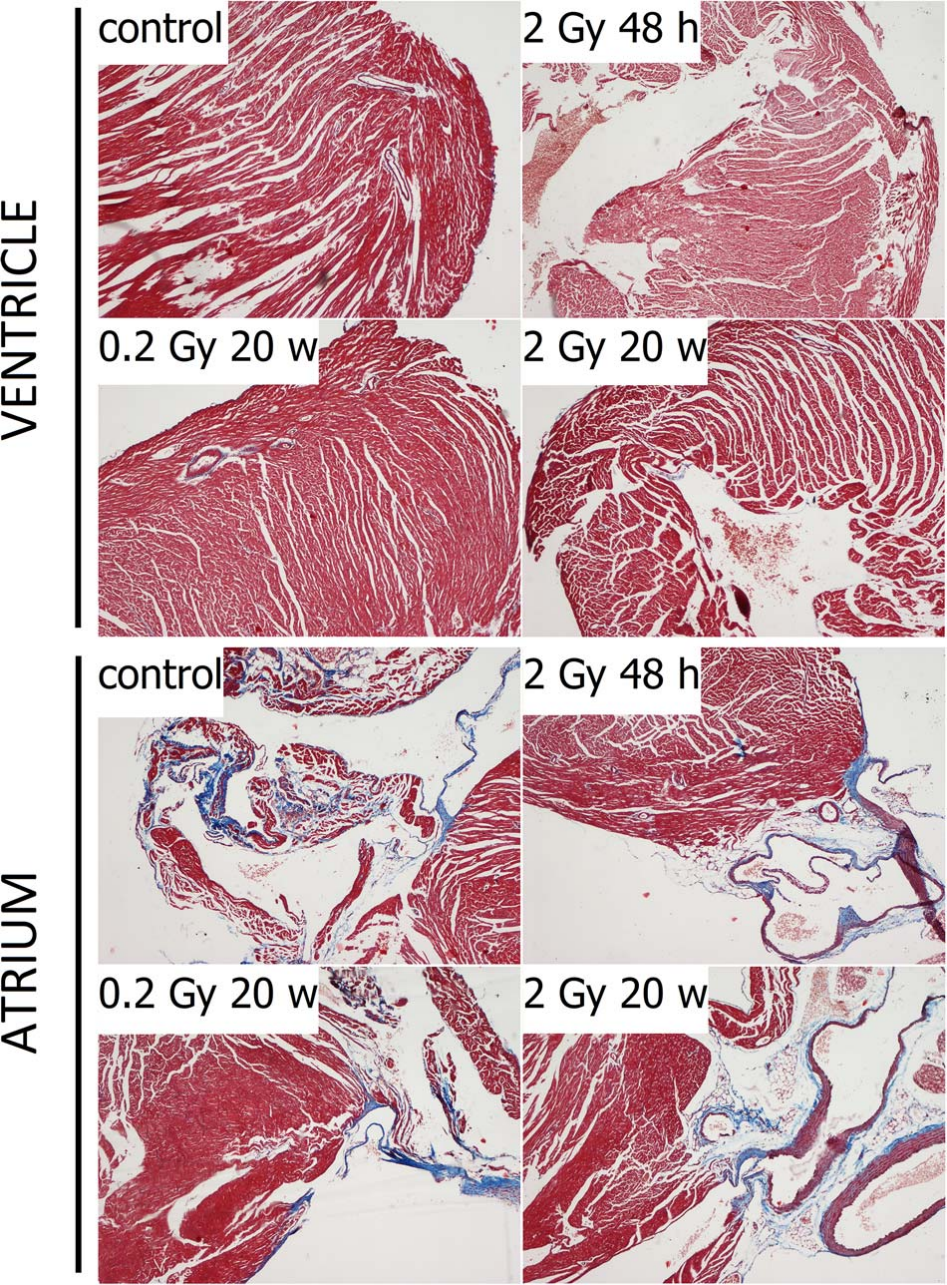
Analysis of collagen content by Masson trichrome staining in atria and in ventricles of control and irradiated mice. Blue, collagen; red, muscle fibers; dark blue, nuclei; light red, blood. Magnification x40.

**Fig. 2.**
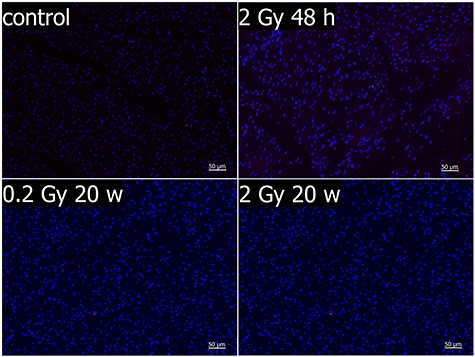
Analysis of apoptotic cells by the TUNEL staining. Blue, nuclei; red, TUNEL-positive cell.

Masson trichrome staining revealed that the majority of collagen fibers were located in the heart atria and the vessels. However, no marked changes in the collagen content in the atria and ventricles of irradiated hearts were observed in either group of mice (Fig.1). Moreover, in all groups of animals, TUNEL staining revealed only single apoptotic cells (3–5 cells per section) in the heart tissue samples (Fig.2), indicating that the implemented doses of radiation did not increase the intensity of apoptotic cell death at the analyzed time points. However, even though the implemented radiation conditions did not affect morphological features of irradiated hearts, marked changes in the profiles of metabolites were observed in corresponding tissues, as described below.

**Fig. 3.**
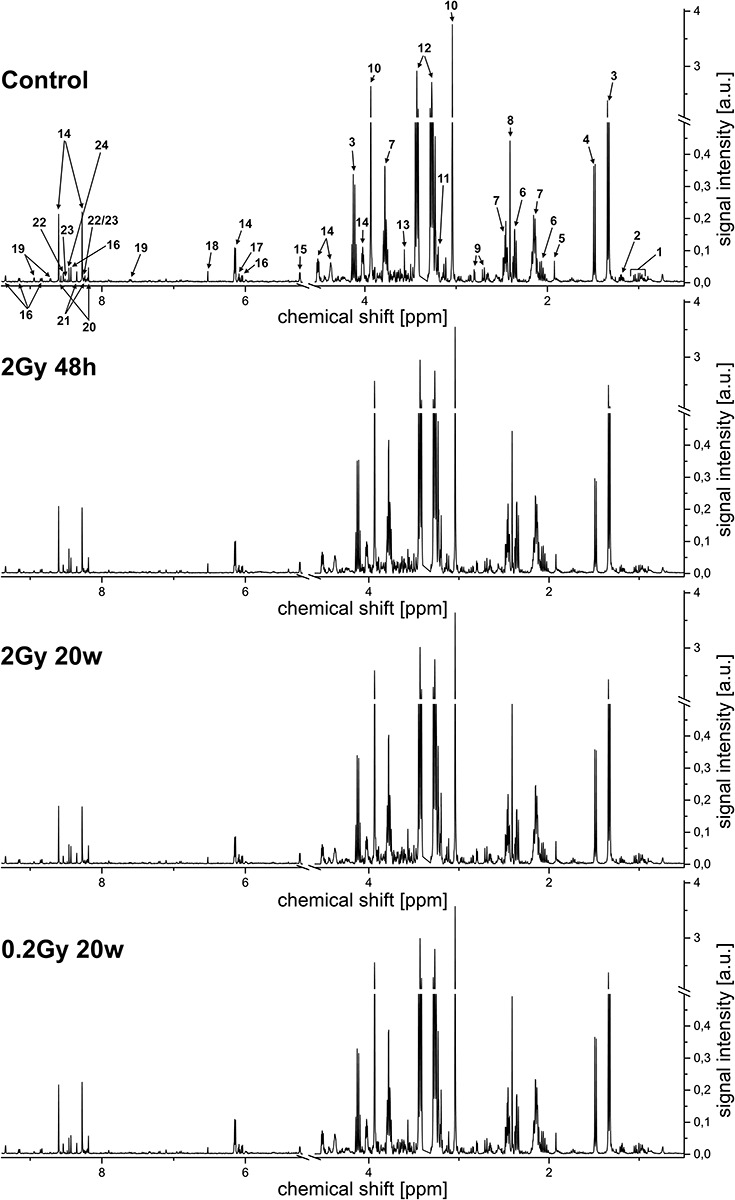
Mean ^1^H NMR NOESY spectra of heart muscle extracts. 1, BCAA (valine (Val), leucine (Leu), isoleucine (Isol)); 2, 3-hydroxybutyrate (3HB); 3, lactate (Lac); 4, alanine (Ala); 5, acetate (Ace); 6, glutamate (Glu); 7, glutamine (Gln); 8, succinate (Suc); 9, aspartate (Asp); 10, creatine (Cre); 11, carnitine (Car); 12, taurine (Tau); 13, glycine (Gly); 14, adenosine monophosphate (AMP); 15, glucose (Glc); 16, nicotinamide adenine dinucleotide (NAD^+^); 17, adenosine (Ade); 18, fumarate (Fum); 19, niacinamide (Nia); 20, inosine monophosphate (IMP); 21, adenosine (Ade); 22, ADP; 23, ATP; 24, formate (For).

**Fig. 4.**
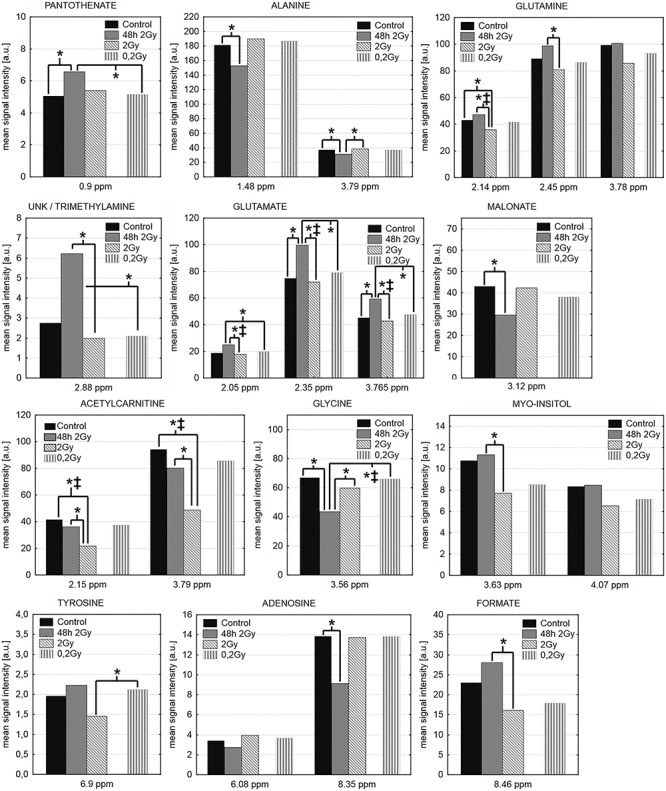
Radiation-induced changes in levels of selected metabolites. In the case of the metabolites with multiple signals in the NMR spectrum all signals are shown, even if no statistically significant change is observed for a particular signal. The statistical significance (*P* < 0.05) is denoted by ^*^ (Mann-Whitney U test) and ‡ (Kruskal-Wallis ANOVA test).

The mean ^1^H NMR NOESY spectra registered for analyzed samples are shown in Fig.3. In general, 44 metabolites were identified (some of them marked in Fig.3). Levels of 33 metabolites were quantified based on the 1D projections of JRES spectra; signals from solvent (water) and impurities (methanol), as well as those with very low signal to noise ratio (due to high quantification error), were excluded from the analyses. The levels of quantified metabolites expressed as peak integral intensities are presented in Table 1. Metabolites whose levels showed statistically significant differences between compared groups of samples are listed in Table 2 and depicted in Fig.4.

**Table 1:** Metabolites identified in NMR spectra; metabolite levels are expressed as peak mean integrals obtained from the JRES spectra ± standard deviation (SD)

Metabolite	Chemical shift^a^	Mean ± SD			
		Control	2Gy 48 h	2Gy 20 weeks	0.2Gy 20 weeks
Pantothenate	0.9 ppm (m)	5.06 ± 1.25	6.57 ± 0.7	5.4 ± 0.85	5.16 ± 0.58
Isoleucine	0.93 ppm (t)	3.7 ± 0.79	4.02 ± 1.15	3.82 ± 1.44	3.73 ± 1.1
	0.99 ppm (d)	6.2 ± 1.69	3.77 ± 1.51	4.63 ± 0.73	5.62 ± 0.86
Leucine	0.94 ppm (m)	4.38 ± 1.64	3.26 ± 1.11	3.9 ± 1.41	4.65 ± 0.94
Valine	0.98 ppm (d)	11.23 ± 2.48	8.49 ± 1.64	10.26 ± 2.22	11.45 ± 2.75
	1.05 ppm (d)	11.14 ± 2.81	8.58 ± 1.66	9.98 ± 2.22	11.15 ± 2.36
Ethanol	1.19 ppm (t)	5.79 ± 3.06	5.83 ± 3.65	9.66 ± 5.14	15.35 ± 9.25
3-Hydroxubutyrate	1.2 ppm (d)	9.06 ± 3.24	9.51 ± 3.67	6.77 ± 3.87	10.54 ± 5.32
Lactate	1.33 ppm (d)	1101 ± 134	1155 ± 184	1059 ± 237	1067 ± 209
	4.12 ppm (q)	150 ± 19	159 ± 27	144 ± 35	146 ± 30
Alanine	1.48 ppm (d)	181.46 ± 9.57	152.64 ± 15.5	189.84 ± 18.57	186.77 ± 34.77
	3.79 ppm (q)	37.45 ± 1.3	31.09 ± 3.24	38.24 ± 2.82	37.06 ± 5.77
Acetate	1.93 ppm (s)	26.51 ± 8.46	22.07 ± 6.21	25.28 ± 5.69	25.37 ± 10.89
Glutamate	2.05 ppm (m)	18.91 ± 4.02	25.02 ± 3.16	17.88 ± 1.03	20.02 ± 2.83
	2.35 ppm (m)	74.85 ± 14.36	99.47 ± 11.69	72.03 ± 2.41	79.23 ± 9.21
	3.765 ppm (q)	45.39 ± 8.38	59.27 ± 6.19	42.68 ± 1.6	47.74 ± 6.61
Glutamine	2.14 ppm (m)	43.17 ± 3.07	47.22 ± 4.26	35.84 ± 0.73	41.72 ± 5.87
	2.45 ppm (m)	89.33 ± 8.69	98.66 ± 9.09	80.89 ± 4.2	86.47 ± 15.35
	3.78 ppm (t)	99.54 ± 12.71	100.56 ± 9.16	85.68 ± 8.71	93.33 ± 20.95
Acetylcarnitine	2.15 ppm (s)	41.7 ± 6.71	36.04 ± 8.36	21.86 ± 3.43	37.56 ± 9.58
	3.19 ppm (s)	94.33 ± 13.8	80.29 ± 18.45	48.86 ± 8.32	85.78 ± 19.05
Succinate	2.4 ppm (s)	308.9 ± 90.8	260.4 ± 50	248.1 ± 35.9	280.8 ± 55
Aspartate	2.65 ppm (q)	16.78 ± 7.99	13.48 ± 4.31	13.46 ± 2.72	14.72 ± 4.09
	2.8 ppm (q)	18.71 ± 8.84	13.99 ± 4.95	14.95 ± 3.87	15.19 ± 4.62
Unk/Trimethylamine	2.88 ppm (s)	2.76 ± 1.72	6.21 ± 3.31	1.98 ± 0.73	2.1 ± 0.89
Creatine	3.0 ppm (s)	1961 ± 200	1954 ± 84	2034 ± 145	1940 ± 221
	3.935 ppm (s)	1264 ± 124	1261 ± 56	1314 ± 99	1252 ± 146
Malonate	3.12 ppm (s)	43.03 ± 7.07	29.53 ± 8.08	42.21 ± 7.65	37.98 ± 11.42
Choline	3.2 ppm (s)	26.61 ± 4.19	21.41 ± 4.59	24.88 ± 4.95	25.94 ± 4.12
Phosphocholine	3.22 ppm (s)	51.03 ± 9.42	61.01 ± 9.82	44.11 ± 8.91	50.03 ± 11.08
Glycerophosphocholine	3.23 ppm (s)	173.7 ± 52.8	230.4 ± 22.5	213.8 ± 17.7	184.9 ± 37.6
Taurine	3.26 ppm (t)	1039 ± 51	1034 ± 36	1002 ± 82	1048 ± 54
	3.43 ppm (t)	1297 ± 72	1286 ± 37	1246 ± 103	1308 ± 73
Glucose	3.49 ppm (t)	26.4 ± 17.94	25.42 ± 6.48	23.94 ± 11	17.01 ± 9.3
	3.54 ppm (m)	60.13 ± 4.19	59.65 ± 2.47	57.39 ± 6.32	61.03 ± 3.41
	3.733 ppm (m)	13.27 ± 9.37	13.46 ± 3.94	11.41 ± 6.55	7.48 ± 5.2
	5.245 ppm (d)	15.11 ± 11.11	15.17 ± 4.99	12.54 ± 5.73	8.98 ± 6.95
Glycine	3.56 ppm (s)	67.12 ± 15.24	43.4 ± 5.95	59.77 ± 6.32	66.04 ± 15.74
Myo-inositol	3.63 ppm (t)	10.76 ± 7.12	11.29 ± 1.73	7.7 ± 1.96	8.51 ± 5.26
	4.07 ppm (m)	8.36 ± 5.10	8.45 ± 1.24	6.52 ± 1.97	7.14 ± 4.84
AMP	4.02 ppm (m)	42.83 ± 10.9	49.32 ± 21.58	53.57 ± 13.32	54.45 ± 17.57
	4.37 ppm (m)	12.21 ± 2.69	15.75 ± 5.75	16.01 ± 4.49	16.75 ± 4.57
	6.14 ppm (d)	29.02 ± 6.62	34.31 ± 12.03	35.79 ± 6.14	36.28 ± 10.18
	8.277 ppm (s)	63.2 ± 14.25	75.63 ± 25.72	79.51 ± 12.13	81.81 ± 22.57
	8.6 ppm (s)	89.88 ± 23.33	106.86 ± 42.6	114.14 ± 24.22	114 ± 37
Creatinine	4.042 ppm (s)	4.59 ± 3.66	5.76 ± 4.21	6.06 ± 4.39	2.17 ± 0.68
NADP^+^/NAD^+^	6.045 ppm (m)	6.61 ± 1.33	5.94 ± 1.3	5.43 ± 2.13	7.17 ± 0.9
	8.18 ppm (s)	20.57 ± 1.79	18.53 ± 2.88	16.73 ± 7.08	21.25 ± 2.46
	8.2 ppm (m)	2.45 ± 0.15	2.79 ± 0.76	2.85 ± 0.83	2.69 ± 0.87
	8.43 ppm (s)	27.16 ± 1.11	24.42 ± 3.93	22.01 ± 8.69	28.28 ± 3.44
Adenosine	6.08 ppm (d)	3.43 ± 0.92	2.74 ± 0.75	3.95 ± 1.9	3.68 ± 1.22
	8.35 ppm (s)	13.88 ± 1.89	9.13 ± 1.58	13.72 ± 8.81	13.83 ± 4.77
Fumarate	6.52 ppm (s)	7.92 ± 2.99	13.55 ± 4.55	13.51 ± 3.79	8.49 ± 2.04
Tyrosine	6.9 ppm (d)	1.96 ± 0.57	2.22 ± 0.49	1.45 ± 0.25	2.12 ± 0.35
Formate	8.46 ppm (s)	23.04 ± 8.05	28.05 ± 6.27	16.03 ± 6.13	17.91 ± 8.67
NADH/NADPH	8.47 ppm (s)	2.33 ± 0.52	4.09 ± 1.46	2.14 ± 0.84	3.57 ± 1.64
ADP	8.53 ppm (s)	10.33 ± 0.85	11.2 ± 1.26	11.67 ± 2.8	13.57 ± 4.36

^a^ NMR signal multiplicity: s, singlet; d, doublet; t, triplet; q, quarter; m, multiplet.

**Table 2:** Radiation-induced changes in levels of metabolites measured by ^1^H NMR spectroscopy; SE, standard error

Metabolite	Chemical shift^a^	Fold change ± SE					
		Control/2Gy 48 h	Control/2Gy	Control/0.2Gy	2Gy 48 h/2Gy	2Gy/0.2Gy	2Gy 48 h/0.2Gy
Pantothenate	0.9 ppm (m)	0.77 ± 0.27^*^	0.94 ± 0.38	0.98 ± 0.35	1.22 ± 0.32	1.05 ± 0.28	1.27 ± 0.28^*^
Alanine	1.48 ppm (d)	1.19 ± 0.18^*^	0.96 ± 0.14	0.97 ± 0.23	0.8 ± 0.16	1.02 ± 0.29	0.82 ± 0.24
Glutamate	2.05 ppm (m)	0.76 ± 0.26	1.06 ± 0.29	0.94 ± 0.33	1.4 ± 0.26^*^^‡^	0.89 ± 0.18	1.25 ± 0.33^*^
Glutamine	2.14 ppm (m)	0.91 ± 0.15	1.2 ± 0.11^*^	1.03 ± 0.22	1.32 ± 0.15^*^^‡^	0.86 ± 0.14	1.13 ± 0.26
Acetylcarnitine	2.15 ppm (s)	1.16 ± 0.46	1.91 ± 0.61^*^^‡^	1.11 ± 0.46	1.65 ± 0.64^*^	0.58 ± 0.24	0.96 ± 0.47
Glutamate	2.35 ppm (m)	0.75 ± 0.23^*^	1.04 ± 0.23	0.94 ± 0.29	1.38 ± 0.21^*^^‡^	0.91 ± 0.14	1.26 ± 0.29^*^
Glutamine	2.45 ppm (m)	0.91 ± 0.17	1.1 ± 0.16	1.03 ± 0.28	1.22 ± 0.18^*^	0.94 ± 0.22	1.14 ± 0.31
Unk/Trimethylamine	2.88 ppm (s)	0.45 ± 0.51	1.39 ± 1.38	1.32 ± 1.38	3.13 ± 2.83^*^	0.95 ± 0.75	2.96 ± 2.83^*^
Malonate	3.12 ppm (s)	1.46 ± 0.64^*^	1.02 ± 0.35	1.13 ± 0.53	0.7 ± 0.32	1.11 ± 0.54	0.78 ± 0.45
Acetylcarnitine	3.19 ppm (s)	1.18 ± 0.44	1.93 ± 0.61^*^^‡^	1.1 ± 0.41	1.64 ± 0.66^*^	0.57 ± 0.22	0.94 ± 0.42
Glycine	3.56 ppm (s)	1.55 ± 0.56^*^	1.12 ± 0.37	1.02 ± 0.47	0.73 ± 0.18^*^	0.91 ± 0.31	0.66 ± 0.25^*^^‡^
Myo-inositol	3.63 ppm (t)	0.95 ± 0.78	1.4 ± 1.28	1.26 ± 1.62	1.47 ± 0.6^*^	0.9 ± 0.79	1.33 ± 1.02
Glutamate	3.765 ppm (q)	0.77 ± 0.22^*^	1.06 ± 0.24	0.95 ± 0.31	1.39 ± 0.2^*^^‡^	0.89 ± 0.16	1.24 ± 0.3^*^
Glutamine	3.78 ppm (t)	0.99 ± 0.22	1.16 ± 0.27	1.07 ± 0.38	1.17 ± 0.23	0.92 ± 0.3	1.08 ± 0.34
Alanine	3.79 ppm (q)	1.21 ± 0.17^*^	0.98 ± 0.11	1.01 ± 0.19	0.81 ± 0.14^*^	1.03 ± 0.24	0.84 ± 0.22
Tyrosine	6.9 ppm (d)	0.88 ± 0.45	1.35 ± 0.63	0.93 ± 0.43	1.53 ± 0.6	0.68 ± 0.23^*^	1.05 ± 0.4
Adenosine	8.35 ppm (s)	1.52 ± 0.47^*^	1.01 ± 0.79	1 ± 0.48	0.67 ± 0.54	0.99 ± 0.98	0.66 ± 0.34
Formate	8.46 ppms (s)	0.82 ± 0.47	1.44 ± 1.05	1.29 ± 1.07	1.75 ± 1.06^*^	0.9 ± 0.78	1.57 ± 1.11

^a^ NMR signal multiplicity: s, singlet; d, doublet; t, triplet; q, quarter; m, multiplet.

Significant changes are denoted with:

^*^(Mann-Whitney U test, *P* value < 0.05),

^‡^(Kruskal-Wallis ANOVA, *P* value < 0.05).

The most frequent differences in levels of metabolites were observed between samples of non-irradiated hearts from control animals and samples of hearts irradiated with the 2 Gy dose and analyzed 48 h after exposure. There were three metabolites significantly upregulated by radiation: pantothenate (an anionic form of pantothenic acid), trimethylamine and glutamate, as well as five metabolites significantly downregulated by radiation: alanine, malonate, acetylcarnitine, glycine and adenosine. On the other hand, there were two metabolites downregulated in samples collected 20 weeks after irradiation (when compared with age-matched non-irradiated controls): glutamine and acetylcarnitine. However, there were several differences in the metabolic profiles of samples of hearts irradiated with 2 Gy and analyzed either 48 h or 20 weeks after exposure. Levels of glutamate, glutamine, acetylcarnitine, myo-inositol, trimethylamine and formate were higher in samples analyzed 48 h after irradiation, whereas levels of glycine and alanine were higher in samples analyzed 20 weeks after irradiation. Interestingly, statistically significant differences were not observed between samples of hearts analyzed 20 weeks after irradiation with the 0.2 Gy dose and age-matched non-irradiated control. However, the level of tyrosine was higher in this group than in samples analyzed 20 weeks after irradiation with the 2 Gy dose. Moreover, several statistically significant differences were observed between samples analyzed 20 weeks after irradiation with the 0.2 Gy dose and samples analyzed 48 h after irradiation with the 2 Gy dose (Table 2 and Fig.4).

**Fig. 5.**
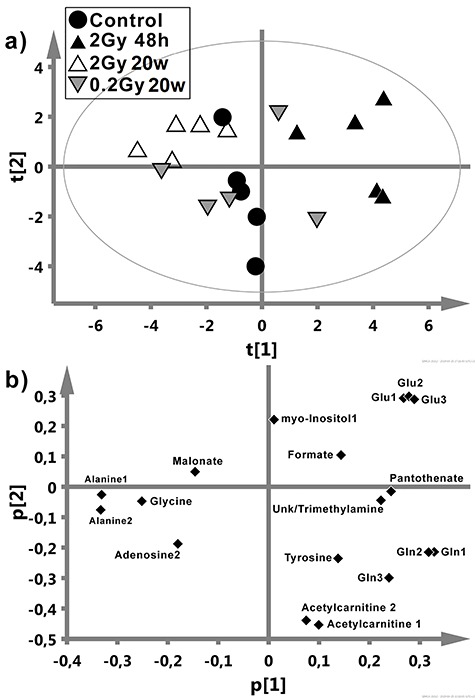
Principal component analysis of NMR data. Shown are score plots (**a**) and corresponding loadings plot (**b**).

Unsupervised PCA was performed based on 18 signals from 12 metabolites listed in Table 2 to reveal differences between analyzed groups of samples. The contribution of variation in the data explained by the model (R2X(cum)) was 71.8%, whereas the predictive ability (Q2(cum)) was 0.19. PCA scores plot (Fig. 5a) showed clear separation of samples analyzed 48 h after irradiation with 2 Gy from controls and samples analyzed 20 weeks after irradiation, which apparently reflected differences observed in supervised analyses. However, separation of controls from samples analyzed 20 weeks after irradiation was not possible by means of unsupervised analysis. Based on the corresponding loadings plot (Fig. 5b) it was possible to identify metabolites responsible for the observed sample clustering in the scores plot (Fig. 5a). The control group was characterized by the highest levels of acetylcarnitine along the second principal component (t [2]). Increased glutamate, pantothenate, unk/trimethylamine as well as decreased alanine, glycine, malonate and adenosine characterized group analyzes 48 h after irradiation with the 2 Gy dose. Samples analyzed 48 h and 20 weeks after irradiation with the 2 Gy dose had an inverse metabolic profile (along t [1]) together with the lowest levels of acetylcarnitine in the latter group (along t [1] and t [2]).

**Fig. 6.**
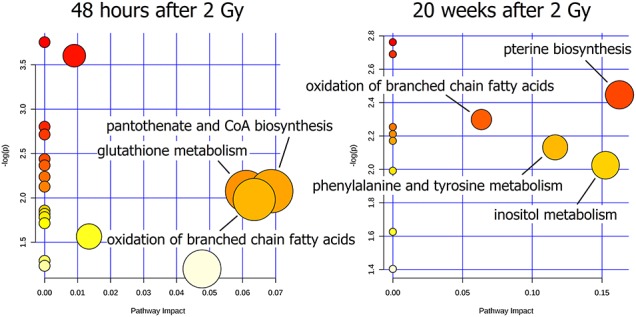
Association of compounds affected by 2 Gy radiation dose with metabolic pathways. Shown are early effects observed 48 h after irradiation and late effects observed 20 weeks after irradiation. Pathways marked as circles are characterized by scores from the enrichment analysis (vertical axes, *P*-value represented by color intensity) and from topology analysis (horizontal axis, pathway impact represented by the size of the circle); an impact score > 0.1 was considered as highly perturbed (in the 2 Gy 20 weeks group: phenylalanine and tyrosine metabolism, inositol metabolism and pterine biosynthesis).

Metabolites affected by radiation in samples analyzed 48 h and 20 weeks after irradiation with the 2 Gy dose (early and late effects, respectively) were assigned to metabolic pathways using the MetaboAnalyst 4.0 software; results aree depicted in Fig. 6. Early changes had an impact on glutathione metabolism, pantothenate and Coenzyme A (CoA) biosynthesis, and oxidation of branched-chain fatty acids (Fig. 6a). Late changes had an impact on oxidation of branched-chain fatty acids, phenylalanine and tyrosine metabolism, inositol metabolism and pterine biosynthesis (Fig. 6b); however, only three later pathways could be considered as significantly perturbed (impact > 0.1).

## DISCUSSION

The molecular mechanisms of the cardiotoxicity of ionizing radiation are multifaceted and many of their aspects remains unclear. Here we aimed to characterize the influence of ionizing radiation on the metabolism of the heart tissue by NMR-based metabolome profiling. According to our knowledge this is the first study investigating the impact of low doses of radiation on heart metabolism. Cardiotoxicity is usually described as a late (or long-term) effect of radiation. Therefore, potential effects induced by a single 0.2 or 2 Gy dose were analyzed 20 weeks after the irradiation. Moreover, to compare the late and early effects of radiation, tissues were analyzed 48 h after exposure to a higher dose as well.

The most distinct radiation-induced changes in the heart metabolism were observed 48 h after irradiation with 2 Gy and involved upregulation of pantothenate and glutamate, as well as downregulation of alanine, malonate, acetylcarnitine, glycine and adenosine. Most of these compounds are involved in energetic pathways, therefore their disturbance may influence the energy production of the heart—an organ with very high energy demand. Pantothenate and acetylcarnitine regulate the biosynthesis of CoA and acetyl-CoA, hence they affect the beta-oxidation of fatty acids [24,25]. An increased level of pantothenate observed 48 h after irradiation could indicate disturbances in the Na^+^ channel gating involved in its transport. In fact, decreased functionality of the cardiac Na^+^/K ^+^-ATPase represents one of the first effects caused by irradiation and leads to intracellular Na^+^ accumulation [26]. Malonate, through malonyl-CoA, also regulates fatty acids metabolism (both synthesis and beta-oxidation) [27]. The decrease in glycine could be linked to its protective role in preserving energy production in mitochondria during stress conditions [28]. Similar glycine depletion was observed in tissues subjected to simulated myocardial hypoxia/reoxygenation [28] or exposed to X-ray radiation [29]. Glutamate is the main amino acid metabolized by the heart in the Krebs cycle. Hence, the increased level of glutamate associated with a decreased level of alanine and glutamine also indicates changes in energy metabolism. Several reports indicate that during ischemia an increase in glutamate uptake with the simultaneous release of alanine and glutamine from the heart occurs [30,31]. Adenosine, a component of ATP is also associated with energy metabolism. Moreover, metabolic changes observed in hearts 48 h after irradiation could be associated with response to oxidative stress. Several previous reports indicated that the increase in glutamate and pantothenate along with glutamine and glycine decrease could result from activation of the antioxidative pathways [25,28,32,33]. Our previous study on the metabolome of cardiomyocytes exposed *in vitro* to the 2 Gy dose and analyzed 48 h after irradiation revealed similar functional significance of such early radiation-induced effects [21]. We observed changes in levels of metabolites associated with induction of oxidative stress (glutamate, GSH, taurine and pantothenic acid), disturbances in energetic pathways (valine, isoleucine, GSH, glycine, threonine and taurine) and membrane damage (phospholipids, choline, glycine and taurine). Importantly, the coherence of data on metabolite profiles registered for living cardiomyocytes irradiated *in vitro* and for tissue extracts from the hearts of animals irradiated *in vivo* was noted in spite of different analytical approaches.

The late effects of radiation on the metabolism of the mouse heart were apparently milder and only two metabolites differentiated hearts analyzed 20 weeks after irradiation with 2 Gy and age-matched controls. These included downregulated acetylcarnitine and glutamine, both associated with energy metabolism and mitochondrial functions. Acetylcarnitine regulates the level of acetyl-CoA and beta-oxidation in mitochondria, and the decrease in its level could suggest intensification of beta-oxidation [24,34]. A reduced level of acetylcarnitine was previously observed as a result of radiation exposure that could indicate impaired mitochondrial functions [35]. Moreover, acetylcarnitine increases membrane stability and protects its permeability [24,36]. Because of its important role in the transport of fatty acids into the mitochondria for subsequent beta-oxidation [37] and ability to protect various cells against oxidative injury [38], it seems that an excessive continuous loss of acetylcarnitine observed in this study as the late effect of irradiation indicates persistent impairment of the antioxidant defense mechanism and energy production through beta-oxidation.

**Fig. 7.**
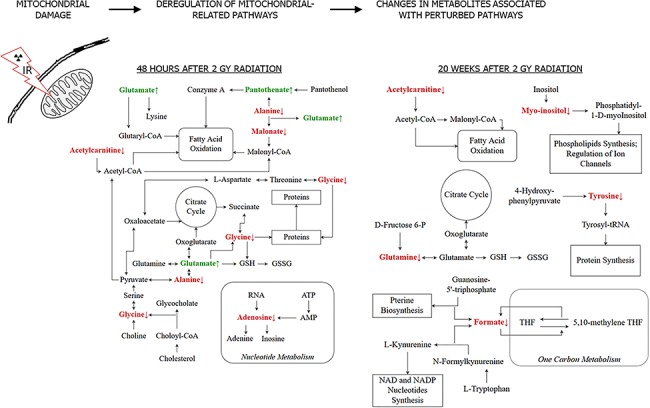
Ionizing radiation leads to metabolic pathways disturbance as a result of mitochondrial damage. Pathways potentially disrupted 48 h and 20 weeks after 2 Gy radiation are presented, based on the results of NMR metabolic measurements. Metabolites found significantly increased and decreased are presented in green and red, respectively, and denoted with arrows. ATP, adenosine triphosphate; AMP, adenosine monophosphate; CoA, Coenzyme A; GSH, glutathione reduced; GSSG, glutathione oxidized; NAD, nicotinamide adenine dinucleotide; NADP, nicotinamide adenine dinucleotide phosphate; THF, tetrahydrofolate; tRNA, transfer RNA (ribonucleic acid).

The analysis of polar metabolites from murine heart extracts allowed us to compare radiation-induced changes observed 48 h and 20 weeks after exposure to a single 2 Gy dose, which corresponded to the early and late effect. Even though apparent differences between the early and late effects were observed at the level of specific metabolites, in both cases affected compounds were associated with similar metabolic pathways including beta-oxidation of fatty acids, production of ATP, oxidative stress response and membrane integrity. Importantly, because these processes are associated with the functioning of mitochondria, observed changes could indicate damage and malfunction of this cellular organelle responsible for energy metabolism. Therefore, it is important to note that damage and malfunction of mitochondria in cardiomyocytes is a frequently described early and late effect of ionizing radiation [39, 40]. Our hypothesis is that ionizing radiation damages the structure of mitochondria, leading to the deregulation of mitochondria-related cellular pathways. This in turn changes the levels of metabolites associated with those pathways, which can be detected by NMR spectroscopy (Fig. 7).

It should be emphasised that the histological tests frequently used to asses toxic effects of radiation (i.e., assessment of collagen fibers and apoptotic cells) did not allow detection of any morphological changes in tissues where NMR spectroscopy enabled detection of radiation-induced changes in metabolic profiles. This indicates that metabolic profiling is a feasible approach enabling the detection of transient molecular changes that precede putative macroscopic changes affecting the structure and function of cardiac tissue after a clinically low (yet radiobiologically high) dose of radiation (i.e., a single 2 Gy dose). However, our study did not reveal any late effects of low-dose irradiation (i.e., a single 0.2 Gy dose) on the metabolic profile of the heart tissue. This could be caused by the limited sensitivity of NMR measurements. On the other hand, the possibility exists that such a small single dose may not affect the function of the heart in a way that will result in metabolic changes detected as the late effects of radiation. Hence, further studies employing more sensitive analytical approaches (e.g., based on mass spectrometry) should be performed to clarify this issue.

Nevertheless, we concluded that metabolome profiling based on NMR spectroscopy revealed several metabolites whose levels were affected in the hearts of mice irradiated with a relatively low dose of radiation (2 Gy). Such differences were observed not only 48 h after irradiation, which corresponded to the direct early radiation effects, but also 20 weeks after irradiation, which corresponded to clinically relevant late effects of radiation. Even though specific metabolites differed between the early and late effects of radiation, they were associated with similar metabolic processes. Moreover, disturbances of processes associated with those metabolites could be correlated with damage and malfunction of mitochondria, which is a general signature of irradiated cells including cardiomyocytes.

## CONFLICT OF INTEREST

The authors declare that they have no conflict of interest.

## FUNDING

This work was supported by the National Science Centre [grant number 2015/17/N/NZ7/04101] and by Maria Skłodowska-Curie Memorial Cancer Center and Institute of Oncology in Warsaw [grant number SN/GW32/2017].
